# The Impact of Learning Approach and Study Habits on Student Performance in an Undergraduate Human Anatomy Course

**DOI:** 10.1007/s40670-025-02424-6

**Published:** 2025-05-26

**Authors:** Andrew R. Thompson, Amulya Vankayalapati, Neal Taliwal

**Affiliations:** Department of Medical Education, University of Cincinnati College of Medicine, 231 Albert Sabin Way, ML 0667, Cincinnati, OH 45267 USA

**Keywords:** Learning approach, Deep learning, Bloom’s taxonomy, Study habits

## Abstract

Developing effective teaching strategies relies on understanding factors that contribute to student learning. Two areas that are commonly considered in this context are study habits and student learning approaches. While study habits can encompass a wide range of behaviors, learning approach is often viewed on two scales: deep and surface. Although learning approach and study habits are interrelated, they are often looked at in isolation and their impact on student learning is not fully understood. With this in mind, we investigated the relationship between learning approach, study habits, and examination performance in a baccalaureate-level human anatomy course. The revised two-factor Study Process Questionnaire was used to determine student learning approach preferences. In addition, an in-house survey was designed to collect data related to the amount of time and distribution effort students used in preparation for examinations. Results indicate that students with a comparatively stronger reliance on a deep approach typically spent more time studying, but other study-related behaviors, such as how study time was distributed, were not consistently correlated with learning approach and learning approach itself was not a strong predictor of examination performance. The most notable finding related to examination performance was that students who increased the amount of time devoted to studying improved significantly, regardless of their learning approach. Overall, these findings highlight the complex nature of student learning and underscore the need to continue refining how learning approach is measured.

## Introduction

As the breadth and depth of information taught throughout various levels of education has grown, so has the exploration of factors that impact student learning and academic achievement. One area that has received considerable attention is student learning approach. The concept of learning approach, first explored in the 1970 s, reflects a student’s motivations and processes for learning new material [1–3]. In most instances, learning approach is viewed on two scales: surface and deep. Students who utilize a deep approach (DA) are driven by an internal desire to learn and employ critical thinking skills. In contrast, students who use a surface approach (SA) are motivated by external pressures, such as an upcoming examination. SA is also more associated with rote memorization, without the same analysis of information that is seen with a DA [4]. Learning approach has been used in a variety of research contexts, but often it is viewed through the lens of predicting academic outcomes [5–13]. Since the DA is believed to result in better learning outcomes, educators often incorporate teaching models that are believed to encourage deep learning, such as problem-based learning and the flipped classroom model [14, 15].

While learning approach aims to measure factors that motivate student learning, on a more fundamental level, the actual methods that students utilize to study can have a major impact on learning outcomes [16, 17]. While study habits can encompass a variety of factors involved in the learning process, previous studies have investigated information about the pace of studying and the studying environment. Although the methods used to collect this data are variable, most often students are asked to complete a survey regarding their study habits. Prior studies found that students earning higher scores were more likely to attend classes in-person, minimize use of third-party online resources, utilize active learning strategies like self-testing, and create a study schedule ahead of time to avoid cramming [18–23].

While the correlation between student learning approach and academic outcomes has been studied, there is little exploration of how learning approach and study habits concurrently influence academic performance. This information is important because when learning activities that encourage deep learning are incorporated, it is expected that students will engage in study habits that reflect this mindset. Accordingly, the aim of this research is to investigate the relationship between learning approach, study habits, and examination performance.

## Materials and Methods

This study includes students who were enrolled in a human anatomy course that is an elective within the Medical Sciences Baccalaureate Program at the University of Cincinnati College of Medicine. Human Anatomy is offered once per year in the Fall semester and is restricted to students who have accumulated the requisite number of credit hours to be classified by the registrar as seniors. The course is taught by two PhD anatomists who also teach in the medical school curriculum. Two student learning assistants are also assigned to the course. These are typically former Human Anatomy students who now attend Medical School at University of Cincinnati College of Medicine. The primary role of learning assistants is to help students during dissection lab and to create practice practical examinations that take place leading up to the real course examinations.

Human Anatomy is designed for students interested in a career in healthcare. As such, the focus is on clinically relevant anatomy and includes the following anatomical regions: back, thorax, abdomen, lower limb, and upper limb. Limited time prevents coverage of the head and pelvic regions. Several different teaching methods are used in the course, including lecture, team-based learning (TBL), and dissection laboratory. The class meets three times per week. There are a total of 19 lecture sessions, 13 dissection laboratories, and five TBL sessions. Lectures and TBLs are scheduled for approximately 1 h, while laboratories are scheduled for 2 h. Laboratory sessions and TBLs have mandatory attendance while lecture attendance is optional (lectures are recorded and made available to students). Dissection groups are comprised of a maximum of four students per human donor. While prosections are used in some anatomical regions (e.g., joints of the limbs), most learning in the laboratory is through hands-on dissection by the students. Outside of scheduled hours, students have unrestricted access to the laboratory for studying purposes.

Assessment of student learning occurs at three points during the course. Lecture material is assessed using multiple-choice examinations administered electronically, while laboratory examinations use a combination of free-response and multiple-choice questions that involve tagging structures on human donors. The two types of examinations typically occur on consecutive days, with the practical scheduled after the lecture examination. Both types of examinations include a combination of higher- and lower-order questions, as defined by Bloom’s taxonomy [24–26]. To provide students with early feedback in the course, back anatomy, which includes only two lectures and two laboratories, is assessed on the first set of examinations and contributes less weight towards the final grade (back lecture exam = 10%, back practical exam = 5%). The remaining sets of examinations each cover two anatomical regions: thorax and abdomen, and upper and lower limbs. These examinations were weighted at 20% for each lecture examination and 15% for each practical examination. The five TBL sessions contribute to the remainder of the grade, each of which is worth 3%. While struggling students may schedule a proctored examination review session with a faculty member, examinations are secure and not returned to students. As part of the normal course quality control process, entire examinations and individual questions are subject to metrics such as Kuder-Richardson Formula 20 (KR-20), comparison of item difficulty and discrimination, as well as review by several content experts.

### Study Design

Students enrolled in the 2021 and 2022 Human Anatomy course were invited to participate in this study. Enrollment in the course was capped at forty students each year. The average age of students was 21 years. Information on gender was obtained from the students’ original college applications. On this form, students were provided the following options regarding their gender identity: female, male, nonbinary, or add another gender (self-identify). In total, male gender was indicated by 27 students and female gender was indicated by 53 students.

The study was explained to each cohort at the conclusion of the first in-class meeting. Briefly, students were told that participation in the study would involve taking several surveys and permit the lead author (ART) to use anonymized course examination grades and college grade point average (GPA). After students were informed of the guidelines, an individual not affiliated with the research project collected consent forms. Data on student participation was not viewed until after course grades were determined at the end of the semester. Survey data was then linked to performance data and anonymized by a third-party individual. Extra credit (3% added to final course grade) was given to students who completed all portions of the research project. If a student wanted to receive extra credit but did not elect to participate in the study, an alternative option was offered where the student could submit narrative reflections on their study approach for each exam. The University of Cincinnati College of Medicine Institutional Review Board approved this study (IRB # 2021–0414).

Student approaches to learning were evaluated using the revised two-factor Study Process Questionnaire (R-SPQ-2 F) [27]. This instrument was chosen because it is a widely used and validated survey [28–30] that can be completed relatively quickly. The R-SPQ-2 F includes 20 statements that participants rate on a 4-point scale. These results are intended to measure learning approach on two scales: surface and deep. Once all participants completed the survey, their responses were coded according to the R-SPQ-2 F guidelines in Biggs et al. [27]. This calculation then provides independent scores for the surface and deep scales that range from 10 to 50. Preference of one approach over another is interpreted by a higher number on a given scale. The instrument was administered twice during the course: once at the start, and again after the final examination.

A custom, in-house survey was also distributed to students immediately following each of the three course examinations. This survey was designed to collect data related to the study habits that students utilized leading up to each examination. The original survey was developed by ART and underwent several revisions based on feedback from former students who took Human Anatomy. The main goal of the review process was to ensure the questions on the survey made sense from a student perspective. Questions related to three themes were the focus of this study: the total amount of time a student reported studying (total hours), the timing of when a student reported to begin studying (start studying), and how each student distributed their study efforts in the time leading up to an examination (study distribution). For total hours, students were provided with a free-response option to estimate the total number of hours they spent studying for the examination. For the start studying variable, students selected one pre-determined description that best reflected when they began to start studying for a given examination. Specifically, students chose from six options, which ranged from one extreme: “I started studying as soon as we started having lectures that would be on this exam”, to the other: “I started studying the weekend before the exam”. For the study distribution variable, students were presented with a drop-down menu to indicate the percent of their study effort for each week leading up to the examination. As a rule, these percentages had to add up to 100%, which reflects their entire study effort for a given examination. For example, a student might indicate they spent 50% the weekend before the examination, 25% the week before the examination, and 25% 2 weeks prior to the examination. While there is some natural overlap between the variables associated with the timing of studying, the goal of asking both was to avoid being misled if a student indicated they started studying early, but in reality they spent the majority of their time studying closer to the date of the examination.

In the analyses, total hours were left as the raw number reported by each student. Responses to the other two variables were coded such that a higher number on each scale equates to patterns less associated with “cramming” (i.e., study neglect followed by a burst of study close to the time of the exam [31]): start studying — (1) = the week or weekend before the examination, (2) = 2 weeks in advance of the examination, and (3) = 3 or more weeks in advance of the examination; study distribution — (1) = 70% effort or more the weekend before the examination, (2) =  < 70% the weekend before the exam, but 100% effort 2 weeks in advance of the examination, and (3) =  < 70% the weekend before the exam, but 100% effort 3 weeks or more in advance of the examination. These parameters were chosen to more equally distribute students within each variable, while still maintaining meaningful groupings that reflect behaviors ranging from cramming near the time of the exam to preparing well in advance. Additional areas evaluated on the in-house survey included lecture attendance, resources utilized, and how and when students utilized lecture recordings. Unfortunately, analysis of these data revealed very little variation in responses among the study participants. As such, the results of these questions did not add anything meaningful to the results and they were not included in the present study.

To evaluate the impact that study habits and learning approach have on student performance, grades from the second (thorax and abdomen) and third (upper and lower extremities) lecture examinations were included in the study. The first examination, covering back anatomy, was not included as it is very different from the other examinations because there is considerably less content covered and there is typically much less variation in student performance (i.e., most students do well on the examination). All learning activities and examination content between the two cohorts of students were identical. In addition to overall examination averages, all questions were assigned a cognitive level using the Blooming Anatomy Tool [32]. These assignments were made by a single individual (ART), who has previously shown a high degree of intra-rater reliability when classifying examination questions [24]. Questions were then divided into higher-order (apply and analyze) and lower-order (knowledge and comprehension) to permit more straightforward comparisons and further reduce observer error [26, 32]. For the second lecture examination, there were 37 lower-order and 14 higher-order questions, whereas the third examination contained 33 lower-order and 21 higher-order questions.

The final area explored was related to student improvement. This involved taking several steps to evaluate whether learning approach and/or changes in the amount of time spent studying influenced how a student performed from the second to third examination. First, the total number of hours studied for each examination were converted to *z*-scores, which resulted in a standardized measurement of how much each student studied relative to their peers. Next, *z*-scores between examinations were compared to identify students who altered their relative study time from the second to third examination. Students were then classified into one of three categories based on this relationship. Students who spent below average hours studying on the second examination and above average hours on the third examination were placed into the “studied more” category. In contrast, students who went from spending above average hours on the second examination to below average hours on the third examination were placed into the “studied less” category. Students who did not change their relative hours studying were placed into the “studied similar” category. This method was employed in favor of simply comparing the raw number of hours reported by each student because there is a tendency for most students to spend more time studying for the third examination simply because it covers a larger volume of information.

### Statistical Methods

Student performance was investigated using average scores for the entire examination and separated into higher- and lower-order question categories. When examination performance was compared between groups, college grade point average (GPA) was used as a covariate. Although GPA may not be ideal when comparing students across institutions, it is a useful metric in this context since all study participants were part of the Medical Sciences Baccalaureate Program and thus took similar courses during their undergraduate career.

Data related to learning approach were analyzed using two methods. The first method used correlations between the raw learning approach scores, examination performance and study habit data. With performance data, partial correlations, controlling for GPA, were used. Study habit data were correlated using Kendall’s Tau-*b*. The second method involved using cluster analysis to categorize study participants using a combination of both their surface and deep learning approach scores. While there are several cluster methods available, a two-step cluster analysis was selected because it accommodates scale data and determines the optimal number of clusters. These classifications were then used as an independent variable when comparing examinations scores using analysis of covariance (ANCOVA). Survey data were investigated according to cluster group membership using Student’s *t*-tests for the total hours reported studying and a Mann–Whitney *U* tests for the start study and study distribution variables. To look at the relationship between student performance and study habits, Pearson correlations were used for total hours studying whereas Kendall’s Tau-*b* was used for the start studying and study distribution variables. Results where *p* ≤ 0.05 were considered statistically significant. All data were analyzed using SPSS version 28 (IBM Corp., Armonk, NY).

## Results

Of the 80 students enrolled over the two semesters investigated in this study, 73 students responded to both study habits surveys and the R-SPQ-2 F. Average deep approach scores were higher than surface approach scores both times the survey was administered. There were no significant changes in survey results from the beginning to end of the course. Results from the survey given at the end of the course was used for the remainder of the study since these are likely a more accurate reflection of the learning approach students employing during the course.

**Fig. 1:**
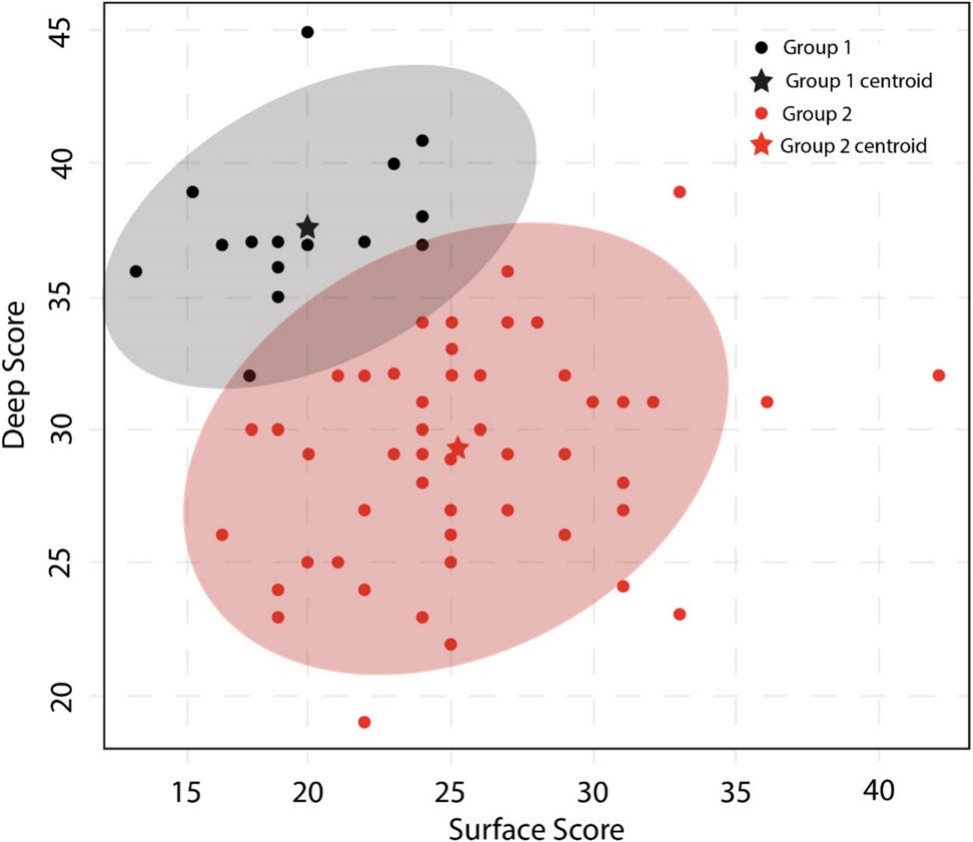
Plot of cluster analysis results with 95% confidence ellipse around group membership

The average surface and deep scores for all study participants were 24.4 ± 4.7 and 31.0 ± 5.1, respectively. There was no significant difference in surface (*p* = 0.95) or deep (*p* = 0.44) scores between the two cohorts of students. Cluster analysis revealed the presence of two discrete groups (Fig. 1). Group 1 (*N* = 16) includes students with an average DA score (37.8 ± 2.9) much greater than their SA score (20.0 ± 3.1). Group 2 (*N* = 57) includes students who, on average, had DA and SA scores that were more comparable (29.0 ± 3.9 and 25.4 ± 4.2, respectively). The remainder of the results will be presented according to each of the main research questions.

### Does Learning Approach Affect Study Habits?

Correlations between the total number of hours spent studying and surface and deep scores revealed no significant relationships for either examination (Table 1). There were no significant relationships between learning approach and when a student started to start or how they distributed their time studying on the second examination. On the third examination, when a student started to study did not significantly correlate with learning approach, but the study distribution had a weak, but statistically significant correlation. In this case, surface scores were significantly negatively correlated (*r* = − 0.253, *p* = 0.008) and deep scores were significantly positively correlated (*r* = 0.235, *p* = 0.014) with study distribution. This relationship indicates that higher deep scores were associated with spreading out studying over a longer time period, whereas higher surface scores were associated with spending a larger proportion of time studying near the date of the examination.

**Table 1:** Relationship between learning approach and study habits

		Surface approach		Deep approach	
		Correlation	*p* value	Correlation	*p* value
Exam 2	Total hours	− 0.16	0.174	0.100	0.397
	Start studying	− 0.04	0.670	0.167	0.073
	Study distribution	0.03	0.750	0.002	0.983
Exam 3	Total hours	− 0.11	0.353	0.168	0.156
	Start studying	− 0.179	0.057	0.081	0.387
	Study distribution	− 0.253	0.008	0.235	0.014

In addition to correlations between learning approach scores, study habits were also compared using group assignments derived from cluster analysis as the independent variable (Table 2). In this instance, the total number of hours studied was significantly different between those in Group 1 compared to Group 2 for both the second (*p* = 0.04) and third (*p* = 0.006) examinations. There were no significant differences in when a student started to study for either examination. For study distribution, there was no significant difference on the second examination, but there was on the third examination (*p* = 0.039).

**Table 2:** Comparison of study habits by cluster group membership

		Group 1	Group 2	*p* value
		Mean (SD)	Mean (SD)	
Exam 2	Total hours	22.6 (9.4)	16.3 (10.5)	0.040
	Start studying	2.13 (0.74)	1.73 (0.85)	0.097
	Study distribution	2.33 (0.72)	2.29 (0.12)	0.864
Exam 3	Total hours	31.0 (18.4)	20.6 (11.3)	0.006
	Start studying	2.32 (0.79)	2.05 (0.88)	0.298
	Study distribution	2.81 (0.40)	2.40 (0.73)	0.039

### What Influence Do Learning Approach and Study Habits Have on Student Performance?

The relationship between learning approach and examination performance was investigated with partial correlations, controlling for GPA (Table 3). For the second examination, there was a weak, but significant negative correlation (*r* = − 0.245, *p* = 0.033) between student performance and surface approach scores. When the examination questions were classified into higher- and lower-order, there were negative correlations between both question levels and surface approach scores and positive correlations with both levels and deep approach scores. However, these patterns were not consistent between examinations and the only significant correlation was a weak relationship between performance and surface approach on the second examination.

**Table 3:** Relationship between learning approach and examination performance

		Surface approach		Deep approach	
		Correlation	*p* value	Correlation	*p* value
Exam 2	Overall	− 0.245	0.033	0.100	0.395
	Higher-order	− 0.187	0.105	0.049	0.674
	Lower-order	− 0.231	0.045	0.105	0.367
Exam 3	Overall	0.031	0.792	0.203	0.079
	Higher-order	0.110	0.343	0.173	0.134
	Lower-order	− 0.031	0.794	0.204	0.077

A similar approach was taken to look at how study habits influenced student performance (Table 4). On the second examination, there were no statistically significant relationships between student performance and the total number of study hours, when a student started studying, or the distribution of time spent studying. On the third examination, there was a significant positive correlation between student performance and total hours spent studying (*r* = 0.300, *p* = 0.011) and study time distribution (*r* = 0.307, *p* = 0.009). These same study habit variables were significantly positively correlated with performance on lower-order question performance, but the results for high-order questions did not reach statistical significance.

**Table 4:** Relationship between study habits and examination performance

		Overall performance		Higher-order		Lower-order	
		Correlation	*p* value	Correlation	*p* value	Correlation	*p* value
Exam 2	Total hours	0.032	0.786	− 0.026	0.823	0.052	0.659
	Start studying	0.144	0.222	0.067	0.571	0.148	0.208
	Study distribution	0.015	0.901	0.104	0.378	− 0.029	0.806
Exam 3	Total hours	0.300	0.011	0.217	0.067	0.268	0.023
	Start studying	0.155	0.193	0.104	0.383	0.159	0.182
	Study distribution	0.307	0.009	0.212	0.073	0.334	0.004

The impact of learning approach on student performance was also investigated by comparing examination results using cluster analysis group membership as an independent variable (Table 5). While individuals in Group 1 consistently outperformed those in Group 2 on both overall examination scores and on higher- and lower-order questions, the differences were generally small, and none were statistically significant.

**Table 5:** Comparison of examination performance by cluster group membership

		Group 1	Group 2	*p* value
		Mean (SD)	Mean (SD)	
Exam 2	Overall	84.7 (8.8)	79.6 (11.7)	0.112
	Higher-order	79.9 (13.9)	75.4 (13.8)	0.285
	Lower-order	86.5 (8.1)	81.1 (12.6)	0.123
Exam 3	Overall	89.8 (8.8)	87.1 (10.5)	0.404
	Higher-order	90.4 (10.8)	87.0 (11.6)	0.330
	Lower-order	89.4 (8.5)	86.7 (12.4)	0.478

### What Factors Contribute to Examination Improvement?

The relationship between relative study hours and examination performance is shown in Table 6. Comparison of overall performance on examination 3 between the groups showed no significant difference in performance. However, when improvement was considered by subtracting examination 3 averages from examination 2 averages, a significant difference was identified (*p* = 0.008). In this case, those who spent comparatively more time studying improved their average by over 16% compared to 7.7% for those who studied similar and − 0.7% for those who studied less. Post hoc Bonferroni test revealed that those who studied more differed significantly (*p* = 0.007) compared to those who studied less, but not compared to those who studied a similar amount (*p* = 0.203). There were no differences in learning approach between the groups.

**Table 6:** Analysis of improvement from the second to third examination

	*N*	Exam 3 avg	Exam 2 to exam 3 avg change	SA	DA
Studied less	13	83.2 (12.6)	− 0.7 (13.2)	23.9 (3.8)	29.9 (5.2)
Studied similar	52	88.6 (9.3)	+ 7.7 (11.4)	24.2 (4.9)	31.5 (4.9)
Studied more	8	87.6 (9.9)	+ 16.2 (12.0)	26.3 (4.1)	29.3 (5.5)
*p* value		0.089	0.008	0.332	0.484

## Discussion

### Learning Approach and Study Habits

Comparing students’ scores from the R-SPQ-2 F and the custom, in-house survey shows that the SA tended to be more associated with less time spent studying and a condensed studying distribution or “cramming,” while the DA was correlated with more hours dedicated to studying with a larger study distribution in preparation for exam three. However, these results should be interpreted carefully as the strength of the correlations were rather weak [33]. Prior studies indicate that the motivations for students who utilize a SA and a DA differ: SA learners are motivated by the external environment, while DA learners have an intrinsic desire to learn [4, 34]. Results from this study are mixed, but data from the third examination do align with this principle as SA scores were associated with organizing study time in a way that suggests the examination timing, which can serve as an external motivator, played a role in shaping study habits. Since a DA is characterized by an internal desire to learn, it is understandable that deep learners’ natural curiosity in the material might lead them to spread their studying out over a longer period, rather than being driven by the pressures of an impending examination. Why this relationship was present on the third examination but not the second is not entirely clear. One possible explanation is that students modified their study habits after the second examination using insight gained on how the material is tested. If this was the case, students who favor a DA may have realized that studying effectively required them to space out their learning over a longer period of time.

There was no significant correlation between learning approach scores and the other two study habit measures: hours spent studying or the start of study. However, the significant difference found in total hours spent studying between cluster group membership is in alignment with one prior study [35]. It has been suggested that this correlation is due to the internal motivation associated with the DA [1, 4]. This motivation and satisfaction from learning may push students to work for longer hours [35]. While the cluster group analysis showed significance, it is curious why the same association was not seen with the raw learning approach scores. As learning approach scores lie on a spectrum, it is possible that correlations to study habits are only detected when students are more reliant on a singular approach. Additionally, as cluster group membership was shown to have a significant relationship with study distribution on the third examination, a greater distribution of studying may have allowed for DA students to spend more hours studying. In contrast to our findings, another study found the SA to be positively correlated with longer time spent studying [36]. It was offered that due to the inefficiency of the SA, students had to spend more time learning the material, employing strategies like rote memorization [1, 36, 37]. Therefore, while there are themes that define each learning approach, how this translates to actual study habits is complex and likely mediated by factors that may not be fully captured in a dichotomous perspective on learning approach.

### Learning Approach and Academic Performance

The current literature on learning approach, as well as the methods of studying that are widely encouraged today in the classroom, greatly favor the DA. Several studies have found that students utilizing a DA tend to perform better on examinations, although the degree to which is variable [5, 6, 8, 13, 38]. Furthermore, educators have been moving towards teaching methods that encourage more student involvement. Models like “problem-based learning,” a “flipped” classroom, have become more common place, boasting improvements in academic performance [39, 40]. These models all involve a level of deeper thinking from students that encourages self-directed learning and discourse. These practices are in alignment with traits that define deep learning, including critical thinking around the material [4]. However, results from the present study were not as absolute. While DA scores were positively correlated with all examination performance markers (overall, lower-order, and higher-order) and SA was negatively correlated with most, there were few statistically significant relationships and those that did exist were not consistent.

The lack of significant findings between examination performance and learning approach in the present study may be attributable to several things. Recently, concerns have been raised surrounding the validity of the R-SPQ-2 F [8, 9, 41–45]. While a variety of potential issues have been proposed, at the core there is uncertainty surrounding whether the instrument provides a valid measurement of learning approach in all populations. For example, [44] found evidence in interview data suggesting that students used a mixed approach that is similar to what was described as an achieving approach in the original Study Process Questionnaire [46] and what others have suggested occurs when students have intention to both memorize and understand [47]. However, inconsistencies in the measurement of the achieving approach resulted in it being removed when the R-SPQ-2 F was developed [27]. LoGiudice et al. [9] also question whether the self-reporting nature of the R-SPQ-2 F provide accurate results, or whether the results are more of a reflection of what students think they should be doing.

Another consideration in the present study is the subject matter itself. While higher-order thinking certainly is and should be a goal when teaching anatomy [48, 49], lower-order concepts serve as key building blocks in being able to apply anatomical knowledge. This idea is central to the theory behind the hierarchical nature of Bloom’s Taxonomy. While this concept has been challenged in some learning contexts [50], when teaching anatomy it is difficult to imagine a scenario where a student can explain complex information without having the fundamental knowledge more associated with lower-order learning processes. This might lead to students to favor what is referred to as the strategic approach [51], which is defined by learners who adjust their approach between surface and deep, depending on the task. This idea is supported by previous studies in anatomy education, which have shown that students tend to migrate towards a strategic approach, and as a result have better academic outcomes [13, 52]. Unfortunately, this specific approach is measured by a different learning approach inventory: Approaches and Study Skills Inventory for Students [51], and is thus not able to be directly investigated in this study. These concerns have implications for interpreting the results in the present study as it is almost inevitable that learning approach was dynamic. This, along with the validity issues pointed out above, could account for why the measurement of learning approach supplied by R-SPQ-2 F was not found to be a strong predictor of academic performance.

### Study Habits and Academic Performance

While most would not dispute that study habits are an important factor in academic success, devising a way to capture how students study is challenging. As such, current literature on study habits and academic success yields mixed results. Some have found that total study time has a positive correlation to academic performance [53, 54]. Intuitively, one would expect that more effort put into learning should result in a better understanding of course material and therefore better academic outcomes. However, some studies indicate that the quality of studying is more important than the quantity of hours [55–57]. Study distribution has also shown to play an important role in learning as those who plan a study schedule tend to have higher achievement [18, 23]. The present study found mixed results. Total hours were not significant predictors of performance on examination two but were significantly associated with student’s performance overall and on lower-order questions on examination three. These results could indicate that as a student progresses through a course, they may better appreciate what study strategies are most beneficial when preparing for examinations. One effective studying habit reported in the literature is spacing out studying rather than “cramming” right before an examination [23, 58–60]. While the results were not absolute, this study found evidence on the third examination that a wider distribution of study time resulted in better performance.

When assessing improvement from examination two to examination three, Table 6 shows the majority of students, even those who did not change their study habits, showed improvement on the third examination. Interestingly, those who altered their approach by dedicating more time to studying improved their academic performance more than twice as much as those who did not alter their effort. This improvement was seemingly independent of learning approach as there was no difference in SA or DA score between these groups of students. Overall, this finding supports what most educators likely already do, which is to counsel students about time management and ensure that the effort they are putting into a class is congruent with their academic goals.

### Limitations

There are several limitations of this study. There is concern that the sample size may have been too small to detect subtle differences in the data that might have otherwise resulted in significant findings with a larger sample. This is somewhat mitigated by the fact that the data were investigated using a variety of approaches and numerous data points were incorporated. Unfortunately, simply increasing the sample size was not possible due to changes in the course structure that precluded the ability to collect additional years of data. The study design was also unable to control for the number of extraneous variables that may impact student performance such as class schedule, mental or physical illness, extenuating life circumstances, or the effort associated with applying to graduate/medical school. These obligations can pull students’ time and attention away from studying and could influence students to take a SA to learning [61] or decreased the amount of time spent studying [62]. Additional obligations can also increase stress and anxiety which has also been shown to decrease academic performance [63]. Another limitation of the study design is the fact that the study habit data collected in this project were self-reported by students. Combating the potential for inaccurate data in this context is extremely difficult as self-reporting is one of the few feasible approaches for collecting study habit data. That said, this study did employ a deliberate effort to mitigate inaccuracies that could stem from lapses in memory by collecting the data directly after each examination.

Another limitation is not accounting for students who have previously been tested with higher-order questions or prior experience learning anatomy, both of which could influence learning approach. Previous studies have shown that students who preferred a DA performed better on both low-level and high-level questions [64]. However, since our study population were senior college students in a rigorous major, it is likely that all students in the study have previously taken a class that utilized higher-order questions that required critical thinking skills. Therefore, while prior experience among the students is likely comparable, its influence in students’ learning approach cannot be completely disregarded.

An inevitable limitation in this study design is that the R-SPQ-2 F measures learning approach at a single point in time and on a dichotomous scale. Study habits and learning approaches are dynamic processes and can vary between courses and even between lectures within the same course [65]. Studies of medical students have shown that initially students preferred a DA, but over time opted in for a SA to learning [10, 66], a finding has been mirrored at the undergraduate level [67]. Another study looking at undergraduate students in China suggests that students change their learning approach based on a variety of factors such as assessment requirements, quality of lecture, student interest, and peer influence [65]. Although learning approach did not change significantly between the two times it was measured this study, this does not account for the fact that students could adjust their approach in real-time based on a wide range of intrinsic and extrinsic factors.

## Conclusion

Understanding the relationship that learning approach and study habits have on student performance is vital to optimize teaching strategies and student learning. Educators typically encourage students to take a DA by designing assessments and learning activities that push students to critically analyze course material. This is done under the assumption that taking this approach will result in better learning outcomes. The present study failed to find a strong or consistent relationship between learning approach and academic performance. While the raw learning approach scores did not predict study habits consistently, those who strongly favored a DA tended to spend more time studying and, in some cases, employ a study routine that was less likely to involve last minute cramming. Although time and study distribution were not significant factors in examination performance on the second exam, they were associated with performance on the third exam and study time was a major factor in how much a student improved on the third exam.

Making sense of these results in terms of how they can be applied to the classroom is complex. Perhaps the clearest results are also those that may seem the most obvious to seasoned educators — when counseling students, it is important to get a good sense of the time they are dedicating to studying to ensure their efforts align with their academic goals in the course. It might appear that the lack of consistent correlation between learning approach and examination performance indicates that learning approach itself is not important in shaping academic outcomes. However, as pointed out above, learning approach is dynamic and there are numerous recent concerns of whether the R-SPQ-2 F is a valid measure of learning approach in all populations. With that in mind, it is our belief that the concept of learning approach has educational value, but viewing it on a dichotomous scale at fixed points in time does not provide an accurate portrayal of how most students approach learning.

## Data Availability

Data used in this study are available upon request.
